# Supplementary material for: Excavation of Molecular Subtypes of Endometrial Cancer Based on DNA Methylation
Source: Genes (Basel). 2022 Nov 13;13(11):2106. doi: 10.3390/genes13112106 (PMC9690162; doi:10.3390/genes13112106)
Supplement: Supplementary file 1 [file genes-13-02106-s001.zip › supplement file/Figure S1.pdf]

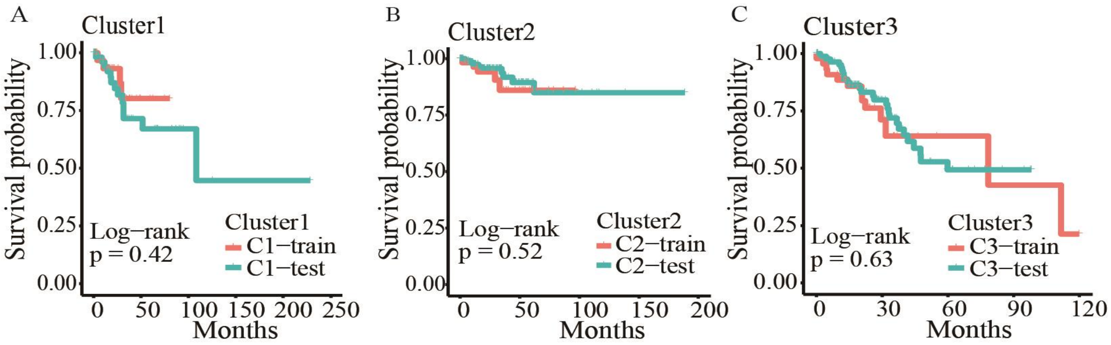

Figure S1. Survival curve of the same label cluster in the train sets and test sets. Log-rank was used to test the statistical significance of the difference. There are no significant differences between any groups.
