# Supplementary material for: Excavation of Molecular Subtypes of Endometrial Cancer Based on DNA Methylation
Source: Genes (Basel). 2022 Nov 13;13(11):2106. doi: 10.3390/genes13112106 (PMC9690162; doi:10.3390/genes13112106)
Supplement: Supplementary file 1 [file genes-13-02106-s001.zip › supplement file/Figure S3.pdf]

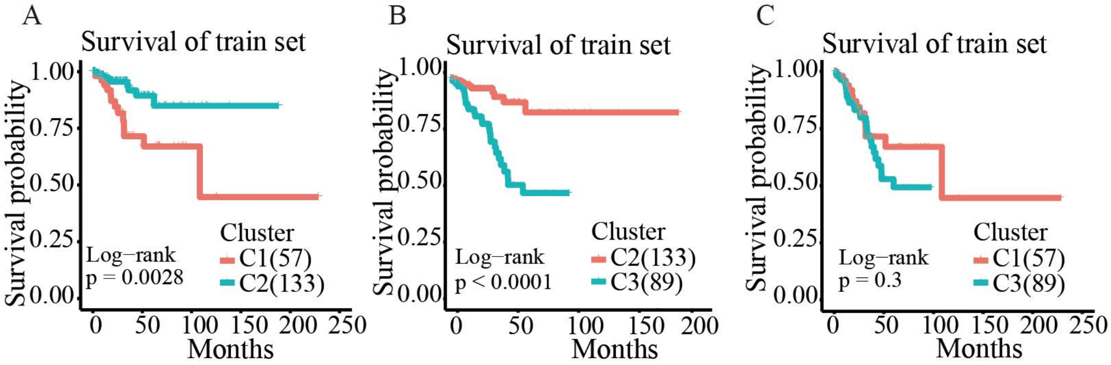

Figure S3. Survival curve for each pair of cluster.Log-rank was used to test the statistical significance of the difference.(A)Cluster 1 and cluster 2 had statistically significant outcomes.(B)Cluster 2 and cluster 3 had statistically significant outcomes.(C)The prognosis of cluster 1 and cluster 3 was not statistically significant.
