# Supplementary figures and images for: Excavation of Molecular Subtypes of Endometrial Cancer Based on DNA Methylation
Source: Genes (Basel). 2022 Nov 13;13(11):2106. doi: 10.3390/genes13112106 (PMC9690162; doi:10.3390/genes13112106)

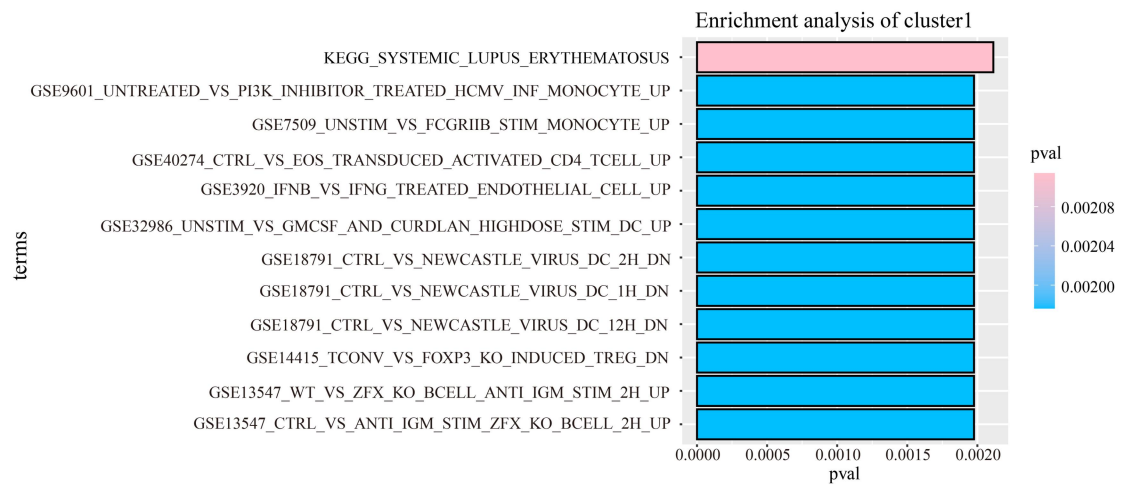

**Figure S2.** Gene enrichment analysis corresponding to specific CpG sites in cluster 1.

Supplement: Supplementary file 1 [file genes-13-02106-s001.zip › supplement file/Figure S2.pdf]

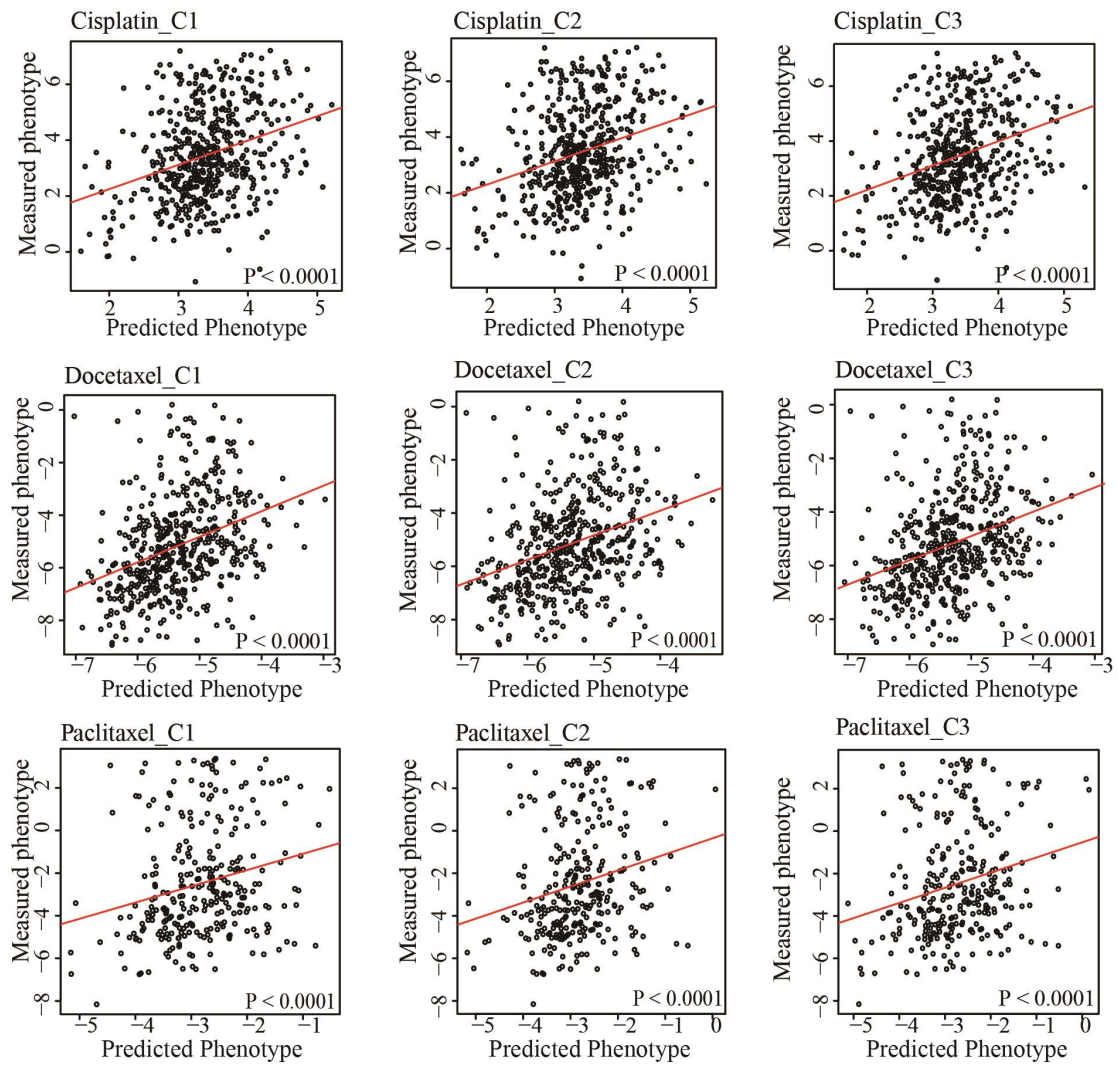

**Figure S4.** Scatter plot shows the level of correlation between the three drugs and each cluster.

Supplement: Supplementary file 1 [file genes-13-02106-s001.zip › supplement file/Figure S4.pdf]
